# In-silico target prediction and pathway analysis of propranolol as a potential therapeutic agent for hepatocellular carcinoma

**DOI:** 10.1371/journal.pone.0333978

**Published:** 2026-02-13

**Authors:** Ishaq Ahmad, Shakeel Ahmad Khan, Muhammad Abu Bakar, Adnan Shakoor, Abdul Wasy Zia

**Affiliations:** 1 School of Fashion and Textiles, The Hong Kong Polytechnic University, Hung Hom, Hong Kong Special Administrative Region, China; 2 Department of Applied Biology and Chemical Technology, The Hong Kong Polytechnic University, Hung Hom, Hong Kong Special Administrative Region, China; 3 Department of Chemistry, University of Agriculture, Faisalabad, Pakistan; 4 Center for Biosystems and Machines, King Fahd University of Petroleum and Minerals, Dhahran, Saudi Arabia; 5 Institute of Mechanical, Process, and Energy Engineering (IMPEE), School of Engineering and Physical Sciences, Heriot-Watt University, Edinburgh, United Kingdom; Guangdong Medical University, CHINA

## Abstract

Hepatocellular carcinoma (HCC) remains lethal despite multitargeted tyrosine kinase inhibitors and immunotherapy, motivating the repurposing of safe, widely available agents. To delineate the anti-HCC potential of propranolol through an in-silico network pharmacology and molecular structure-based study, 70 intersecting potential anti-HCC targets were retrieved from the SwissTargetPrediction and GeneCards databases. Protein–protein interaction (PPI) analysis identified a network of 64 interconnected nodes exhibiting a high average node degree of 9.84, highlighting target centrality. Subsequent hub analysis isolated nine pivotal proteins (SRC, EGFR, CCND1, JAK2, ERBB2, PARP1, CDK4, CDK2, CHEK1) with degree centrality values exceeding 23.2, more than twice the network average. Gene Ontology and KEGG enrichment analyses underscored robust involvement in oncogenic pathways, including PI3K–Akt, MAPK, and immune checkpoints. Molecular docking revealed strong binding affinities of propranolol toward key kinases, notably JAK2 (–8.14 kcalmol^-1^), ERBB2 (–7.80 kcalmol^-1^), EGFR (–7.76 kcalmol^-1^), and CDK2 (–7.44 kcalmol^-1^). Molecular dynamics simulations confirmed the complex stability, with RMSD values stably maintained below 4.5 Å over 100 ns simulations. The sustained hydrogen-bond occupancy ranged from 30% to 68% per trajectory, corroborating stable ligand engagement. Collectively, these factorial results provide compelling evidence that propranolol may interact with core oncogenic kinase cluster and potential modulation of the critical signaling cascades implicated in HCC pathogenesis. Collectively, these computational findings support the hypothesis that propranolol possesses the molecular characteristics of a viable therapeutic candidate for HCC, thereby substantiating the need for rigorous experimental and translational investigation to validate its clinical potential.

## Introduction

Hepatocellular carcinoma (HCC), which accounts for 75–85% of primary liver cancers, remains the third leading cause of global cancer mortality, with an estimated 30,090 deaths projected in the US in 2025 alone. HCC mortality rates have increased from 3.65 to 5.03 per 100,000 persons between 2006 and 2022, and are projected to continue to increase, reaching approximately 6.39 per 100,000 persons by 2040, underscoring the urgent need for effective interventions [1]. In recent decades, a spectrum of therapeutic modalities, including surgical resection [2], liver transplantation [3], locoregional therapies, and systemic treatments, have been extensively utilized in HCC management [4]. However, access to these definitive interventions is limited to approximately one-third of patients [5]. In addition, early-stage detection remains paramount, as timely intervention significantly enhances prognosis and patient survival. Unfortunately, the majority of HCC cases are diagnosed at intermediate or advanced stages, rendering curative options such as resection and transplantation unfeasible [6]. The propensity for vascular invasion and subsequent intrahepatic and extrahepatic metastases contributes to poor outcomes and elevated post-therapy recurrence rates.

Sorafenib, a multi-tyrosine kinase inhibitor, targeting multiple kinases including VEGFRs, PDGFR, and Raf kinases, has emerged as a frontline systemic agent for advanced HCC, albeit conferring only a modest survival benefit of approximately three months [7]. Conversely, other tyrosine kinase inhibitors, such as lenvatinib, inhibit VEGFR1–3, EGFR1–4, PDGFRα/β, KIT, and RET, demonstrating non-inferior overall survival compared to sorafenib but with distinct toxicities, including hypertension and proteinuria [8]. Given the limited effective therapeutic options, it is imperative to explore novel pharmacological strategies to improve clinical outcomes. Moreover, the protracted timelines (~10–15 years), exorbitant costs ($2.0–2.6 billion per approved drug), and high attrition rates associated with de novo drug discovery have impeded the rapid translation of novel agents into clinical practice.

Given the pressing clinical challenges posed by HCC, drug repurposing is a vital emerging therapy that accelerates drug development by leveraging existing drugs with established safety profiles to discover new treatments more quickly, cost-effectively, and with reduced risk, offering promising solutions to redefine therapeutic strategies and improve patient outcomes in this devastating disease [9–12]. Propranolol, a well-established non-selective beta-adrenergic receptor blocker, has been successfully repurposed for several cancer types, demonstrating antitumor effects by inhibiting proliferation, angiogenesis, and metastasis [13,14]. In cancers such as melanoma and cervical carcinoma, propranolol has shown promising results by modulating the key signaling pathways involved in tumor growth and survival [9,15,16]. Although preliminary studies indicate that propranolol may reduce HCC risk and improve prognosis by targeting β-adrenergic receptors and inducing apoptosis in HCC cells [17], the detailed molecular targets and signaling pathways underlying its therapeutic effects in HCC remain unknown. Therefore, there is a pressing need to explore and understand the mechanism of action of propranolol in targeting multiple oncogenic pathways [18,19].

**Fig 1:**
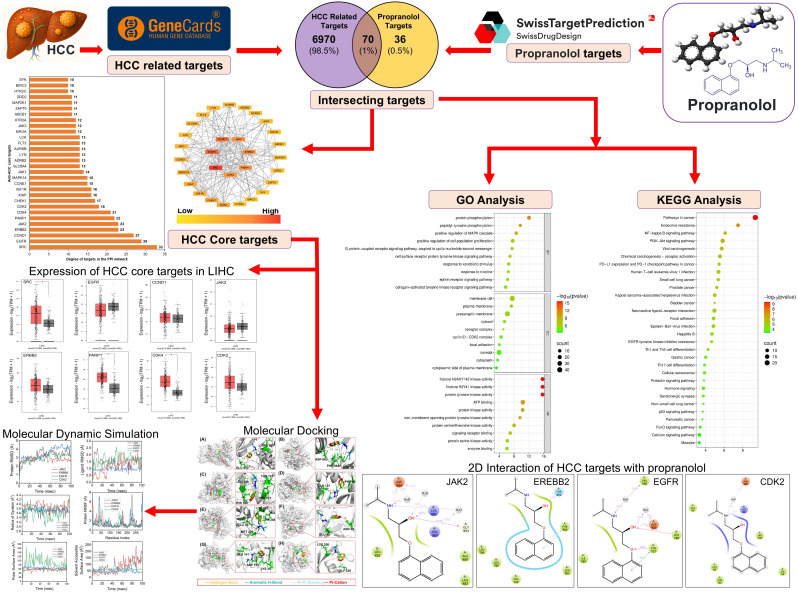
Schematic workflow for predicting the therapeutic potential of propranolol in HCC using network pharmacology, molecular docking, and molecular dynamics simulations. Key steps include target identification, pathway enrichment analysis, expression profiling, and in silico validation of propranolol–target interactions.

Network pharmacology combined with molecular docking has emerged as a powerful strategy. By integrating drug and disease targets data and leveraging large-scale public biomedical databases, network pharmacology constructs comprehensive drug–target–disease interaction maps that reveal key molecular mechanisms and potential therapeutic targets across complex biological systems [20]. In the context of HCC, this integrated approach enables the elucidation of propranolol’s potential molecular targets and signaling pathways, which are otherwise challenging to dissect experimentally. Therefore, in this study, we employed network pharmacology and molecular docking to comprehensively analyze and predict the key targets and pathways through which propranolol may exert antitumor effects in HCC, providing a rational basis for its repurposing as a novel therapeutic agent for HCC. The Schematic workflow of this study has been presented in the Fig 1 given below.

## Materials and methods

Several software and online tools, including databases, were used in this study, as shown in S1 Table.

### Prediction of potential targets of propranolol

The online database SWISS Target Prediction was accessed on May 28, 2025, to predict the potential targets of “propranolol”. The *Homo sapiens”* species was selected before running the program for drug prediction [21]. The predicted potential targets were selected based on a probability greater than zero.

### Determination of HCC related targets

The “GeneCards^®^: The Human Gene Database” was accessed on May 28, 2025, to identify potential targets related to HCC [22]. Four scientific keywords for HCC, namely hepatic cancer, hepatoma, hepatic carcinoma, and hepatocellular carcinoma, were used to obtain HCC-related targets with a relevance score ≥ 5.

### Intersecting targets related to HCC and propranolol

The online tool VENNY 2.1 was used on May 28, 2025, to obtain the intersecting targets between HCC and propranolol potential targets [23].

### Protein-protein interaction (PPI) analysis

An online tool, STRING 12.0, was used to analyze the protein-protein interactions by setting the STRING to *“Homo sapiens”* multiple protein mode with a medium confidence score of 0.400 [24]. PPI network data were obtained from STRING in the form of tab-separated values (tsv) file format and further analyzed using Cytoscape 3.10.3 version. [25]. Potential anti-HCC core targets were identified based on the degree of connection in the PPI network.

### Molecular complex detection (MCODE) analysis

The MCODE Plug-in of Cytoscape was used to identify the critical modules in the PPI network of the core key target genes. The conditions for MCODE analysis were set as follows: find clusters in the whole network, degree cutoff = 2, node score cutoff = 0.2, k-core = 2, and Maximum Depth = 100.

### Enrichment analysis

Gene Ontology (GO) functional enrichment and Kyoto Encyclopedia of Genes and Genomes (KEGG) pathway analyses were conducted on 64 key targets using the DAVID bioinformatics resource (accessed on June 04, 2025) to elucidate their biological functions and associated signalling pathways [26]. Gene Ontology (GO) terms were systematically classified into cellular components (CC), biological processes (BP), and molecular functions (MF). The most significantly enriched terms and pathways were visualized through an enrichment dot bubble chart using an advanced bioinformatics platform (Figs 4 and 5, respectively) [27], enabling an integrative and intuitive representation of the results. Statistical significance was rigorously assessed via the classical hypergeometric test, with multiple hypothesis correction performed using the Benjamini–Hochberg procedure, applying a stringent adjusted *p*-value cutoff of <0.05, to ensure robust and reliable enrichment results.

### Expression of core anti-HCC targets

The GEPIA database (accessed on June 11, 2025) was used to examine the expression of the top nine anti-HCC targets in liver hepatocellular carcinoma (LIHC) (GEPIA, 2022 (Gene Expression Profiling Interactive Analysis)) [28].

### Molecular docking

The anti-HCC core targets and drug ligands were prepared using Maestro 14.4 from the Schrödinger Suite 2025 version 1. Molecular docking was performed using Glide within the same suite [29].

#### Anti-HCC core targets preparation.

The top nine potential anti-HCC core targets (SRC, EGFR, CCND1, JAK2, ERBB2, PARP1, CDK4, CDK2, and CHEK1) were retrieved from the Protein Data Bank (PDB) [30]. The crystal structures were processed using the Protein Preparation Wizard (PPW) in the Schrödinger Suite 2025−1. Structural refinement involved the correction of side chains, assignment of bond orders, addition and optimization of hydrogen atoms, and adjustment of protonation states to pH 7.4 to simulate physiological conditions. Missing residues and loops were reconstructed using the Prime module, and disulfide bonds were verified and restored. Water molecules located beyond 5 Å from the protein were removed, and the metal ions were modeled with zero-order bonds to maintain the coordination geometry. A secondary filtration step eliminated water molecules within 3 Å of the non-water hydrogen atoms to optimize the binding site environment.

Energy minimization was performed using the OPLS_2005 force field, applying restraints to maintain the backbone rigidity while allowing side-chain flexibility. The minimization converged at a heavy-atom RMSD threshold of 0.30 Å, ensuring structural integrity and readiness for subsequent docking studies.

#### Ligand preparation.

The ligand, propranolol, was retrieved from the PubChem database in the 3D SDF format. Ligand preparation was conducted using the LigPrep module of the Schrödinger Suite 2025−1. The protocol involved the addition of explicit hydrogen atoms, generation of ionization states at a physiological pH (7.4), and stereochemical optimization. Subsequent energy minimization was performed using the OPLS_2005 force field to refine the ligand’s three-dimensional geometry, ensuring an energetically favorable conformation for molecular docking studies.

#### Receptor grid generation.

Receptor grids were generated using the Glide module in the Schrödinger Suite 2025−1 to define the docking search space. The grid center and dimensions were based on the coordinates (x, y, and z) of the co-crystallized ligands to fully encompass the active site. For proteins lacking bound ligands, we identified potential binding pockets using the Sitemap tool, which predicts and scores possible ligand-binding regions by analyzing the physicochemical properties of the protein surface. These predictions guided the selection of grid centers and dimensions, ensuring an accurate representation of the binding site. This approach enabled the precise delineation of the receptor environment, facilitating reliable docking simulations.

#### Glide docking of prepared anti-HCC core targets.

Molecular docking was performed using the Glide module in Schrödinger Suite 2025−1 employing the Extra Precision (XP) mode. Pre-prepared receptor grids and the propranolol ligand were used as inputs, with all other parameters set to default. XP docking applies an anchor-and-grow sampling algorithm with an exhaustive ligand conformational search and post-docking minimization using the OPLS_2005 force field. The receptor was treated as rigid, whereas ligand flexibility was fully sampled to explore diverse binding poses. Docked poses were ranked using the GlideScore function, which integrates multiple energetic terms to estimate the binding affinity. Detailed information on the top nine potential anti-HCC core targets in the PDB database and grid docking parameters in molecular docking are provided in S2 Table. The docked complexes were further visualized using Maestro and PyMOL.

### Molecular dynamic simulation

Molecular dynamics (MD) simulations were conducted using Desmond 2020.1 (Schrödinger) to investigate the stability and interaction profiles of four protein-ligand complexes, JAK2-Propranolol, ERBB2-Propranolol, EGFR-Propranolol, and CDK2-Propranolol, selected based on their superior predicted binding affinities. The OPLS3e force field was employed to accurately describe molecular interactions. Each protein-ligand complex was explicitly solvated using the SPC water model within an orthorhombic periodic boundary box extending 10 Å beyond the complex in all dimensions.

The initial structures were prepared using the Protein Preparation Wizard, which included the assignment of bond orders, addition of missing side chains and hydrogen atoms, optimization of protonation states at physiological pH, and energy minimization. The system setup was completed using the System Builder module.

The simulation systems underwent stepwise equilibration and energy minimization under isothermal-isobaric (NPT) conditions for 12 ns, maintaining a temperature of 310 K and a pressure of 1 atm. Temperature control was implemented using a Nose–Hoover thermostat chain, while pressure regulation employed the Martyna–Tuckerman–Klein barostat chain. Long-range electrostatics were treated using the Particle Mesh Ewald (PME) method with a 9 Å real-space cutoff for Coulomb interactions. Integration was performed using a 2 fs timestep.

MD simulations were performed for 100 ns to ensure thorough sampling and assess complex stability. The trajectories were analyzed for structural stability and dynamic behavior using metrics including the root mean square deviation (RMSD), root mean square fluctuation (RMSF), protein-ligand interaction fractions, ligand contact mapping, radius of gyration (rGyr), solvent-accessible surface area (SASA), and polar surface area (PSA).

### Molecular mechanics to determine free binding energies

Molecular mechanistic evaluations employing the Generalized Born Solvent Accessibility (MM-GBSA) framework were conducted to estimate the binding free energies (ΔG_bind) of the complexes of propranolol with anti-HCC targets. These calculations were performed using the Prime MM-GBSA module of the Schrödinger Suite 2025−1, which couples molecular mechanics energies (E_MM) with the OPLS4 optimized force field for accurate liquid-phase simulations. The VGSB implicit solvent model was selected due to its superior capacity to generate solvent-free energy estimates within the generalized Born formalism compared with chloroform-based and other solvation models. Binding free energies for each protein–ligand system were subsequently derived using the standard ΔG_bind computational expression.


$${\Delta \text{G}}_{\text{Bind}}={\text{G}}_{\text{Complex}}-({\text{G}}_{\text{Protein}}+{\text{G}}_{\text{Ligand}})$$

where G_complex_ represents the energy of the receptor-ligand complex, G_protein_ is the energy of the receptor, and G_ligand_ is the energy of the unbound ligand.

## Results

### Identification of intersecting targets

**Fig 2:**
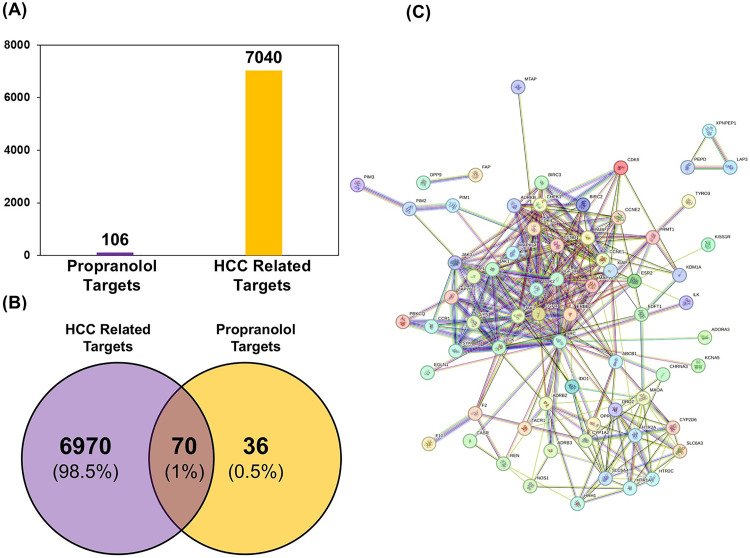
Network pharmacology identification of propranolol–HCC intersecting targets and their protein–protein interaction landscape. **(A)** Potential propranolol targets and HCC-related targets, **(B)** Venn Diagram showing intersecting potential anti-HCC targets, **(C)** STRING Protein – Protein Interaction (PPI) network of potential 70 anti-HCC targets of propranolol.

A total of 106 potential propranolol targets with probability scores > 0 were identified using the SwissTargetPrediction tool and saved for further analysis Further, 7040 potential HCC-related targets with a relevance score of ≥ 5 were retrieved from the GeneCards database as shown in Fig 2(A). Among these retrieved targets of propranolol and HCC-related targets, a total of 70 intersecting targets were retrieved using the Venny 2.1 online tool, as shown in the Venn diagram in Fig 2(B). The resulting 70 intersecting targets were considered potential anti-HCC targets of propranolol.

### PPI network analysis

The potential 70 anti-HCC targets of propranolol were subsequently submitted to the STRING database (Search Tool for the Retrieval of Interacting Genes/Proteins) to construct a comprehensive protein-protein interaction (PPI) network. The analysis was restricted to *Homo sapiens* to ensure species-specific relevance of the results. The resulting PPI network comprised 70 nodes representing the individual targets and 319 edges denoting the predicted functional associations between them Fig 2(C). The network exhibited an average node degree of 9.11, indicating that each target (protein), on average, interacts with approximately nine other targets within the network. Furthermore, the network demonstrated an average local clustering coefficient of 0.611, reflecting a relatively high tendency of the targets to form tightly inter-connected clusters. Notably, the observed number of edges (319) substantially exceeded the expected number of edges (126) for a random network of comparable size, suggesting a significant enrichment of interactions among these targets and highlighting their potential coordinated role in the molecular mechanisms underlying HCC and the pharmacological effects of propranolol.

**Fig 3:**
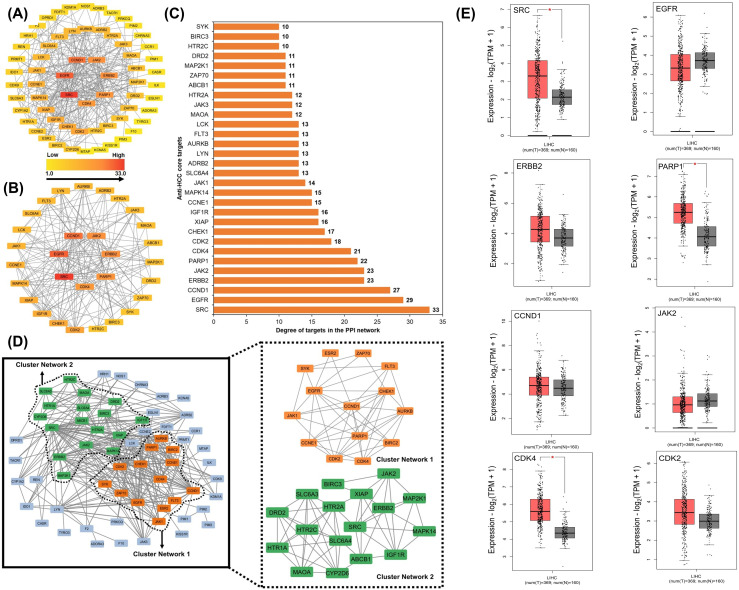
PPI-based hub and module analysis of predicted anti-HCC targets and validation of core target expression in LIHC. **(A)** PPI network of the potential anti-HCC targets, **(B)** PPI network of the top 30 anti-HCC targets with a degree greater than the average (9.84). In both networks **(A and B)**, the transition from yellow to red indicates a degree shift from low to high for each node. **(C)** Thirty anti-HCC core targets in the hub network ranked by DC > 9.84 (DC = Degree centrality). **(D)** MCODE network clusters within the PPI network of the core targets. **(E)** Expression of the top eight anti-HCC core targets in LIHC (red and grey boxes represent tumor and normal cells, respectively) (LIHC = Liver hepatocellular carcinoma).

The PPI network data obtained from STRING were imported into Cytoscape software to enhance visualization and facilitate more detailed network analysis. During this process, disconnected nodes and targets without any interactions within the network were removed to focus on the integrated network components. After exclusion of these isolated nodes, the refined network consisted of 64 nodes interconnected by 315 edges, yielding an average number of neighbors per node of 9.84, indicative of a moderately dense interaction landscape, as shown in Fig 3(A). Network topology analysis revealed a diameter of 5, representing the longest-shortest path between any two nodes, and a radius of 3, denoting the minimum eccentricity within the network. The characteristic path length was 2.25, which reflects efficient connectivity. Additional network metrics included a clustering coefficient of 0.48, suggesting moderate local clustering among proteins; a network density of 0.156, indicating the proportion of actual connections relative to all possible connections; a heterogeneity of 0.74, reflecting variability in node connectivity; and a centralization of 0.379, demonstrating a moderate degree of central nodes dominating the network. These parameters collectively characterize the structural and functional organization of the PPI network, providing insights into the potential biological interactions relevant to the effects of propranolol on HCC-related targets.

Furthermore, 30 nodes with DC values > 9.84 were separated and designated as the core anti-HCC targets of propranolol shown in Fig 3(B). The 30 core anti-HCC targets and their degrees of centrality (DC) are shown in Fig 3(C). Owing to their DC values, the top nine nodes, SRC, EGFR, CCND1, JAK2, ERBB2, PARP1, CDK4 CDK2, and CHEK1, were considered hub targets for further analysis. Among these nine hub targets, seven (SRC, EGFR, JAK2, ERBB2, CDK4, CDK2 and CHEK1) are kinase proteins while two targets (PARP1 and CCND1) belong to non-kinase proteins.

### MCODE cluster analysis of the PPI network of anti-HCC key targets

The PPI network comprising 64 key anti-HCC targets was subjected to topological cluster analysis using the MCODE plugin in Cytoscape, which identified densely interconnected subnetworks within complex biological networks. Network analysis identified two highly connected clusters as shown in Fig 3(D). Cluster 1 (14 nodes, 49 edges, score = 7.538) is enriched with key cell-cycle and DNA repair regulators, with CCND1 and EGFR showing the highest connectivity (degree = 11), followed by PARP1 (10), CDK2 and CHEK1 (9). Cluster 2 (17 nodes, 56 edges, score = 7) comprises signaling, metabolic, and survival-related proteins, with HTR2A (degree = 9) and SRC, CYP2D6, SLC6A4 (degree = 8) as central hubs. The degree patterns indicated that Cluster 1 centered on oncogenic kinases and cell cycle control, whereas Cluster 2 integrated neurotransmitter signaling, metabolism, and anti-apoptotic pathways. Notably, the top nine hub targets were localized within these clusters, underscoring their potential centrality and therapeutic significance in HCC.

### Expression of anti-HCC core targets in LIHC

The expression profiles of the top nine anti-HCC targets (SRC, EGFR, CCND1, JAK2, ERBB2, PARP1, CDK4, and CDK2) were quantitatively analyzed in liver hepatocellular carcinoma (LIHC) and corresponding normal tissue samples using the GEPIA database, as shown in Fig 3(E). Differential expression analysis revealed statistically significant alterations in the expression levels of these targets between LIHC and normal samples. These findings substantiate a strong association between the dysregulation of these eight core targets and the pathogenesis and progression of LIHC.

### Gene ontology (GO) enrichment analysis of anti-HCC targets of propranolol

**Fig 4:**
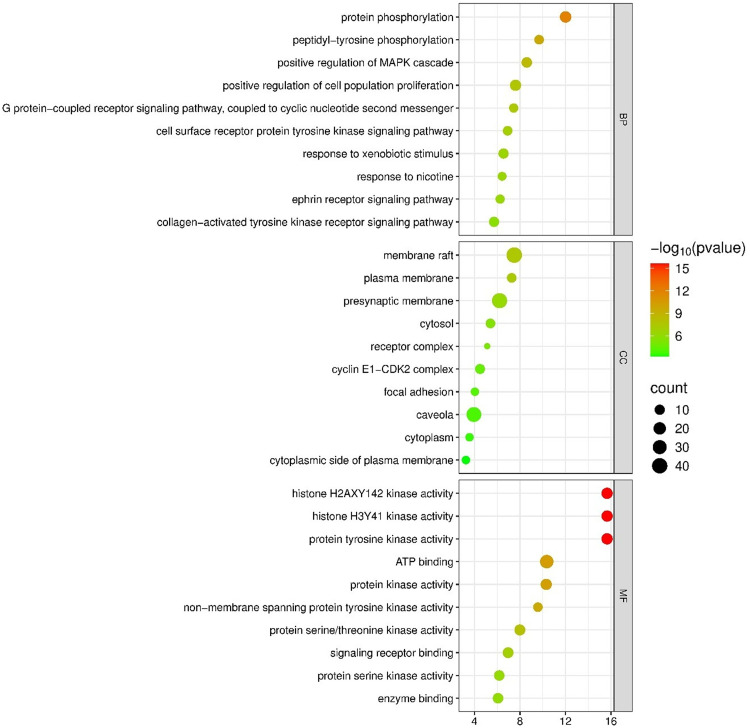
Top 10 GO enrichment analyses of the 64 core anti-HCC targets.

GO enrichment analysis of the 64 anti-HCC targets revealed their significant involvement in key biological processes (BP), cellular components (CC), and molecular functions (MF), as shown in Fig 4. With respect to biological processes, the targets were predominantly associated with protein phosphorylation, peptidyl-tyrosine phosphorylation, and MAPK cascade activation, underscoring their roles in kinase-driven signal transduction and regulation of cell proliferation.

Additional enrichment of protein–coupled receptor signaling and xenobiotic response pathways highlights adaptive cellular mechanisms. In terms of cellular components, the enrichment of anti-HCC targets of propranolol was observed in membrane microdomains, including membrane rafts and plasma membrane, as well as cytosolic and receptor complexes, indicating localization within critical signaling hubs. Among the molecular functions, kinase activities, notably protein tyrosine and histone kinase functions, were strongly enriched alongside ATP-binding and receptor interaction capabilities, reflecting the target’s central roles in phosphorylation and signal modulation. This integrative analysis delineates the molecular framework through which propranolol exerts therapeutic effects in HCC.

### KEGG pathway enrichment analysis of anti-HCC targets of propranolol

**Fig 5:**
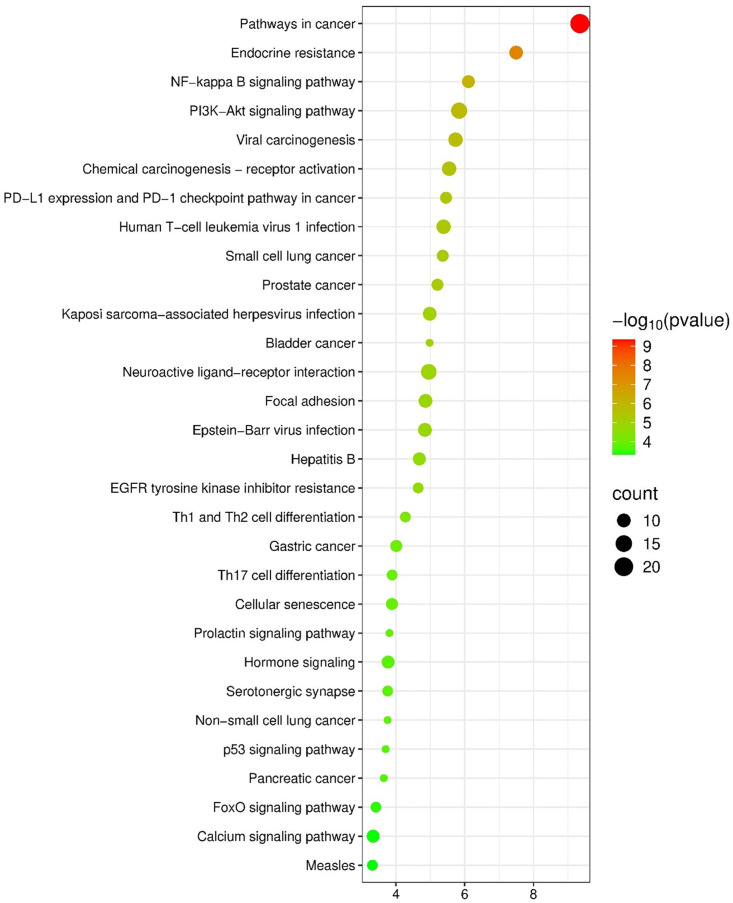
Top 30 most significant KEGG pathways of anti-HCC core targets of propranolol.

KEGG pathway enrichment analysis of 64 core anti-HCC targets of Propranolol identified 78 significant pathways (*p* < 0.05), with the top 30 pathways highlighted in Fig 5. The analysis revealed predominant enrichment in cancer-related and key signal transduction pathways.

The “Pathways in cancer” category showed the highest significance (–log_10_(*p value*) > 9) and encompassed the largest number of targets. Other enriched oncogenic pathways included PI3K–Akt signaling, endocrine resistance, viral carcinogenesis, PD-L1/PD-1 checkpoint, and multiple cancer-specific pathways (e.g., lung, prostate, and gastric cancers), underscoring propranolol’s engagement with core tumorigenic networks.

Signal transduction pathways such as NF-κB, EGFR tyrosine kinase inhibitor resistance, p53, FoxO, focal adhesion, and calcium signaling were also significantly enriched, reflecting the modulation of proliferation, apoptosis, and stress responses.

Immune-related pathways, including Th1/Th2/Th17 differentiation and viral infection pathways (e.g., HTLV-1, Epstein–Barr virus, and Hepatitis B), suggest immunomodulatory potential. Additional pathways related to cellular senescence, hormone signaling, and neurotransmission further illustrate the diverse functional scope of propranolol.

Bubble plot visualization encodes pathway significance (color gradient) and target gene count (bubble size), highlighting the most relevant biological processes studied.

### Molecular docking

**Fig 6:**
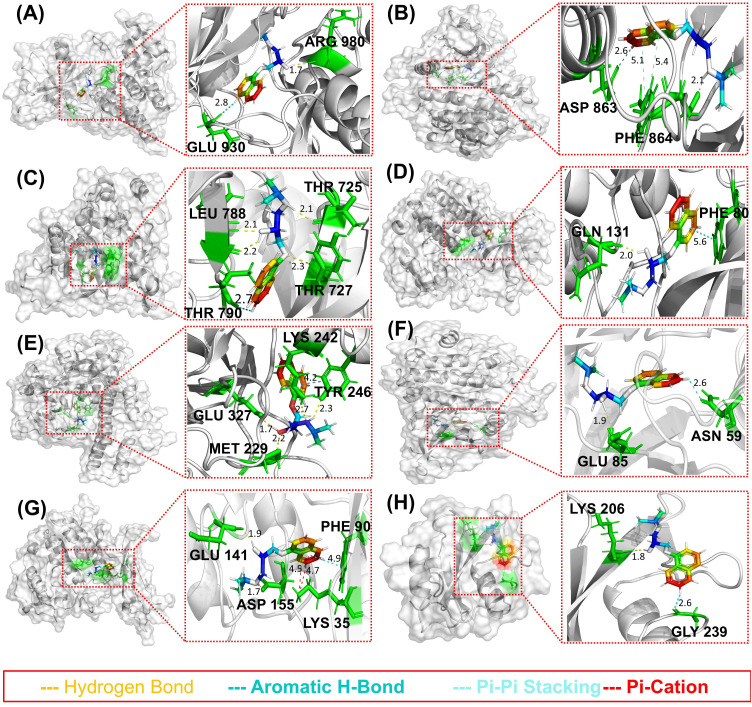
Molecular docking results for (A) JAK2, (B) ERBB2, (C) EGFR, (D) CDK2, (E) PARP1, (F) CHEK1, (G) CDK4, and (H) SRC.

Molecular docking of propranolol against nine anti-HCC targets revealed notable binding affinities and specific hydrogen bonding interactions (Table 1). Propranolol exhibited the strongest predicted binding to JAK2 (PDB ID: 2B7A, binding energy: –8.14 kcal/mol), ERBB2 (3PP0, –7.80 kcal/mol), EGFR (1XKK, –7.76 kcal/mol), and CDK2 (1G5S, –7.44 kcal/mol), suggesting that these kinases are the primary targets. Key hydrogen bonds were observed with catalytically relevant residues, such as ARG 980 (JAK2), PHE 864 (ERBB2), and LEU 788, THR 725, TYR 727 (EGFR), as shown in Fig 6, indicating the potential for stable ligand–receptor interactions. Lower binding energies were observed for CCND1, CDK4, and SRC, indicating weaker interactions. Collectively, these results highlight the multi-target binding profile of propranolol, with a preferential affinity for kinases implicated in HCC pathogenesis.

**Table 1:** Molecular docking results of propranolol, Sorafenib and Lenvatinib against the nine anti-HCC targets.

Receptor	PDB ID	Residue involved in H-Bonding	Docking Score		
			Propranolol	Sorafenib	Lenvatinib
JAK2	2B7A	ARG-980 (1.7 Å)	−8.13	−5.83	−2.09
ERBB2	3PP0	PHE-864 (2.1 Å)	−7.79	−8.17	−7.71
EGFR	1XKK	LEU-788 (2.1 Å), THR-725 (2.1 Å)	−7.76	−9.12	−10.38
CDK2	1G5S	GLN-131 (2.1 Å)	−7.44	−7.0	−6.37
PARP1	2RCW	GLU-327 (1.7 Å), LYS-242 (2.7 Å), MET-229 (2.2 Å),	−6.80	−3.87	−6.15
CHEK1	2AYP	GLU-85 (1.9 Å)	−6.41	−5.15	−4.78
CDK4	6P8E	ASP-155, GLU-141 (1.9 Å)	−3.70	−3.12	−3.05
SRC	1A08	LYS-206 (1.8 Å)	−3.66	−3.13	−3.37
CCND1	6P8E	GLU-75, LYS-180, THR-184	−3.57	−1.4	−3.38

Further, Sorafenib and Lenvatinib were also docked against anti-HCC targets and docking scores have been given in the Table 1. Notably, propranolol has shown more binding affinity with the anti-HCC targets with only exception of EGFR for which Sorafenib and Lenvatinib have more affinity, however, it has been well established that EGFR has become resistant against these Sorafenib and Lenvatinib.

### Molecular dynamic simulation

**Fig 7:**
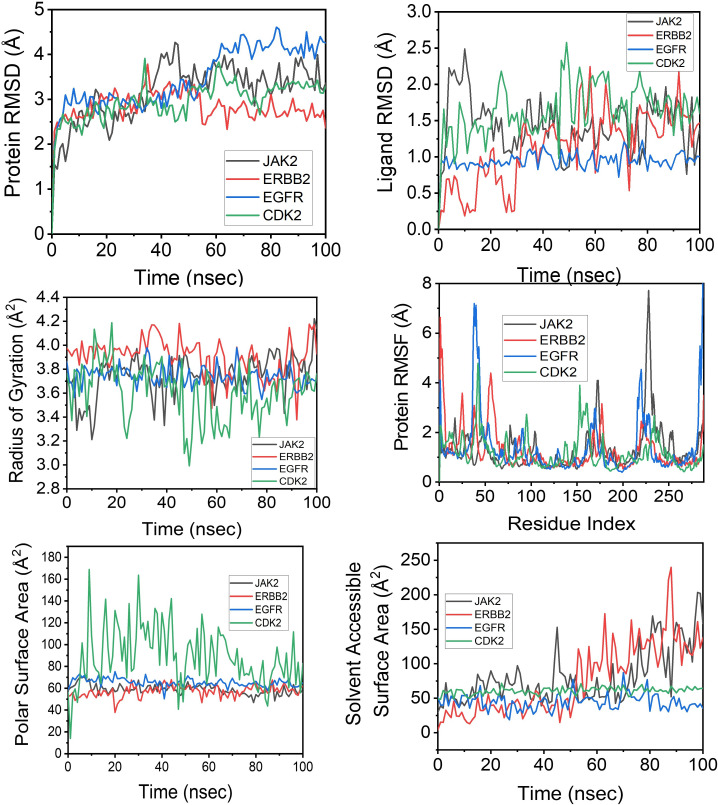
Molecular dynamics simulation analysis of protein-ligand complexes for JAK2, ERBB2, EGFR, and CDK2 over 100 nanoseconds. **(A)** Protein backbone RMSD indicating overall structural stability, **(B)** Ligand RMSD depicting ligand-binding stability, **(C)** Radius of gyration demonstrating protein compactness throughout the simulation, **(D)** Per-residue protein RMSF highlighting regions of flexibility, **(E)** Polar surface area showing solvent-exposed polar regions, and **(F)** Solvent-accessible surface area reflecting protein surface exposure.

**Fig 8:**
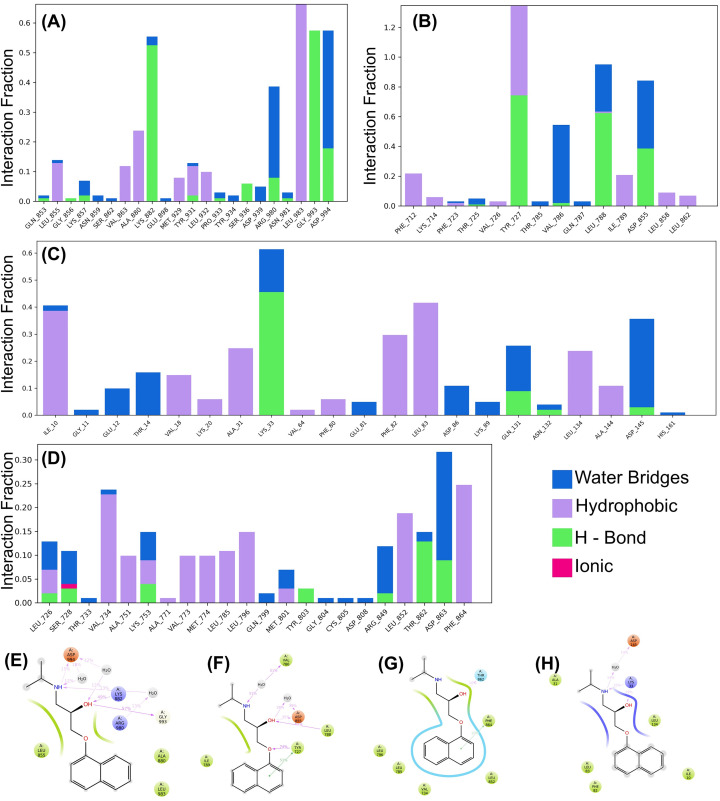
Interaction analysis of protein-ligand complexes highlighting key residue interactions over simulation trajectories. Bar plots (A-D) depict the interaction fractions of various residues categorized by interaction types for JAK2, EGFR, CDK2, and ERBB2. Panels (E-H) illustrate detailed 2D interaction maps for representative ligand-binding for JAK2, EGFR, CDK2, and ERBB2, respectively.

Owing to their higher binding affinities, four complexes were selected for MD simulation analysis. The results for all four complexes are shown in Fig 7(A–H) and 8(A–H). Molecular dynamics simulations of all four complexes over a 100 ns period demonstrated consistent and stable molecular conformations, as substantiated by the root-mean-square deviation (RMSD) values associated with the Cα backbone (Fig 7A). For the JAK2-propranolol complex, the rapid RMSD increase during the first 10 ns indicated the structural reorientation and adaptation of the protein to the propranolol ligand. The RMSD fluctuated between 2.5 and 4.0 Å, which is consistent with the acceptable standards for stable binding [31]. For the ERBB2-Propranolol complex, the RMSD increased rapidly within the first 10 ns and stabilized early at approximately 2.5–3.0 Å. Compared to the other complexes, ERBB2 showed the least fluctuation, maintaining a steady RMSD (~2.8 Å), indicating a relatively stable protein-ligand complex throughout the trajectory, supported by a stability of under 4 Å of RMSD in the literature [32,33]. Similarly, there was an increase in RMSD up to ~3.0 Å within 10 ns for the EGFR – propranolol complex, which then consistently fluctuated and increased to ~4.5 Å. The RMSD fluctuations up to 4.5 Å fall within the acceptable values of stability for EGFR protein, as reported by other researchers [34]. For the CDK2 – propranolol complex, an increase in RMSD was observed for the initial 15 ns, followed by stabilization at approximately 3.0–3.5 Å in the remaining trajectory. A relevant study confirmed that RMSD deviations of up to approximately 4 Å during molecular dynamics simulations of CDK2 protein-ligand complexes are considered acceptable and indicative of stable binding [35].

Interaction analysis of all four complexes revealed that hydrogen bonds, water bridges, and hydrophobic interactions were predominant, with fewer ionic interactions throughout the simulation. Notably, hydrogen bonds were critical for maintaining the binding conformations of the complexes. This was corroborated by two-dimensional (2D) ligand-protein interaction diagrams, which illustrated that significant residue interactions occurred upon binding (Fig 8).

In addition, the structural stability of the top four protein–ligand complexes, JAK2–propranolol, ERBB2–propranolol, EGFR–propranolol, and CDK2–propranolol, was further interrogated through detailed Gibbs free energy (ΔG_Bind_) calculations. Our analysis demonstrated that the predominant stabilizing forces within these complexes arise from Coulombic (ΔG_Bind_Coulomb), van der Waals (ΔG_Bind_vdW), and lipophilic (ΔG_Bind_Lipo) interactions as shown in the Table 2. Conversely, covalent bonding (ΔG_Bind_Covalent) and solvation free energy (ΔG_Bind_SolvGB) were found to detract from complex stability. Importantly, all four complexes exhibited markedly favorable binding free energies, underscoring their strong and persistent protein–ligand associations. These findings suggest that propranolol forms highly stable and energetically favorable interactions with the key oncogenic targets, supporting its potential efficacy in molecular targeting strategies.

**Table 2:** Binding free energies for the propranolol-target complexes were calculated using MM-GBSA.

Energies (kcal/mol)	Docked complexes			
	JAK2-Propranolol	ERBB2-Propranolol	EGFR-Propranolol	CDK2-Propranolol
ΔG_Bind_	−36.15	−43.71	−47.53	−40.68
ΔG_Bind_Lipo	−18.81	−23.37	−16.90	−19.23
ΔG_Bind_vdW	−41.83	−37.37	−37.75	−37.39
ΔG_Bind_Coulomb	−3.70	−8.72	−12.19	−9.76
ΔG_Bind_Hbond	−1.20	−1.11	−2.10	−0.74
ΔG_Bind_SolvGB	22.55	19.28	12.90	23.15
ΔG_Bind_Covalent	6.84	8.55	8.53	4.17

## Discussion

HCC poses complex therapeutic challenges due to its molecular heterogeneity, frequent late-stage diagnosis, and poor response to existing drugs such as sorafenib, which extends survival modestly by approximately three months [7]. In this context, our integrative network pharmacology and molecular simulation analysis revealed that propranolol, a non-selective beta-adrenergic receptor blocker, may hold multifactorial therapeutic promise distinctly superior to many current options owing to its broad target engagement and functional impact on pivotal HCC-associated pathways. In addition, Propranolol has superior physicochemical characteristics that may enhance therapeutic efficacy in HCC compared to Sorafenib and Lenvatinib. The comparative physicochemical characteristics of Sorafenib, Lenvatinib and Propranolol are given in S3 Table.

In this *in silico* investigation, we combined target predictions obtained from SwissTargetPrediction with a dataset of Propranolol and HCC-related genes derived from GeneCards, resulting in the identification of 70 overlapping candidate proteins. Graph-theoretic interrogation of the protein–protein interaction network isolated nine high-centrality nodes, SRC, EGFR, CCND1, JAK2, ERBB2, PARP1, CDK4, CDK2, and CHEK1, whose average degree (DC = 23.2) exceeded the network average by more than twofold, underscoring their pivotal roles in HCC biology. Notably, each of these hubs was independently associated with aggressive clinicopathological behavior and therapeutic resistance, highlighting their collective relevance as pathogenic drivers and potential therapeutic leverage points. For example, elevated c-SRC activity markedly accelerates tumorigenesis and promotes epithelial-to-mesenchymal transition (EMT) [36], which is directly correlated with poor patient survival. Notably, lenvatinib-resistant HCC cells frequently exhibit heightened c-SRC expression; however, pharmacological inhibition effectively restores drug responsiveness [37]. Similarly, the EGFR pathway is significantly implicated, with amplification of UBE2Q1 enhancing β-catenin-EGFR-PI3K–Akt signalling [38], thereby fostering aggressive tumor behavior. Moreover, pronounced EGFR activation characterizes lenvatinib-resistant cells, which regain sensitivity upon treatment with EGFR inhibitors such as erlotinib [39]. Cyclin D1 (CCND1) amplification or overexpression is recurrently documented in human HCC, correlating with inferior histological grades and prognostic outcomes [40]. Targeted Cyclin D1 degradation via combination therapy with lonafarnib and sorafenib reverses sorafenib tolerance through ATG3-mediated autophagic mechanisms [41]. The JAK2 axis is similarly implicated, with elevated MEX3C-JAK2/STAT3 signalling enhancing metastatic capacity and signifying poor prognosis [42], whereas disruption of the CSF3R-AS/CSF3R/JAK2/STAT3 positive-feedback loop reverses sorafenib resistance [43]. ERBB2 (HER2) amplification, detectable by liquid biopsy, is associated with aggressive metastatic progression [44], and increased EGFR/ERBB2 copy number gains post-lenvatinib treatment indicate HER2’s role in tyrosine kinase inhibitor (TKI) resistance [45]. The PCNA-PARP1 axis further supports malignant progression [46], with PARP1 overexpression closely linked to adverse clinical outcomes and identified as a key factor underlying sorafenib resistance, which is counteracted by combining sorafenib with the PARP inhibitor, olaparib [47]. Dysregulated CDK4 signalling, specifically through the DYNLL1/ILF2-CDK4 pathway, promotes rapid cell cycle progression and tumor growth [48], with CDK4/6 inhibition (e.g., palbociclib) demonstrating synergistic efficacy with regorafenib or sorafenib in resistant models [49]. Concurrently, CDK2 upregulation driven by OLA1 overexpression or SMYD3-mediated mechanisms involving MMP2 markedly enhances intrahepatic metastasis and sorafenib resistance [50]. Finally, checkpoint kinase 1 (CHEK1) is significantly overexpressed at both the mRNA and protein levels, independently serving as an adverse prognostic marker in HCC, highlighting its potential as a critical therapeutic target [51,52].

**Fig 9:**
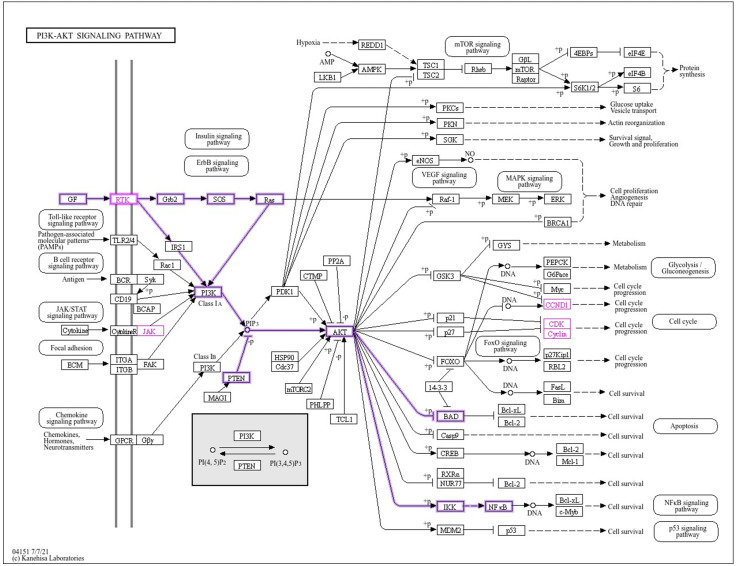
Anti-HCC core targets of Propranolol mapped within the PI3K–AKT signaling pathway (Pathway ID: hsa04151) from KEGG pathways. The PI3K–AKT cascade is annotated to highlight six key targets of Propranolol implicated in hepatocellular carcinoma (HCC): EGFR (RTK), ERBB2 (RTK), JAK2, CCND1, CDK4, and CDK2. Shown in magenta, these proteins govern upstream signaling and cell cycle progression. Their collective positioning within this oncogenic network illustrates Propranolol’s potential for multi-target interference and supports its therapeutic repurpose in HCC.

GO profiling demonstrated that the propranolol-interactable target set is preferentially localized to membrane microdomains and receptor supracomplexes and is embedded in kinase-centric programs, protein and peptidyl-tyrosine phosphorylation, MAPK cascades, and GPCR signaling, consistent with the circuitry that sustains HCC [53,54]. Concordant KEGG enrichment prioritized “Pathways in cancer,” PI3K–Akt, NF-κB, EGFR-TKI resistance, PD-1/PD-L1 checkpoint regulation, and viral carcinogenesis, reflecting contemporary pathway maps and resistance axes described for HCC [53]. Mechanistically, hyperactivation of the PI3K–Akt pathway and aberrant immune checkpoint signaling are recognized as correlates of poor or heterogeneous responses to immune checkpoint blockade in HCC, underscoring the relevance of these nodes to clinical outcomes [55]. Moreover, NF-κB remains a principal driver of inflammation-mediated hepatocarcinogenesis and interacts bidirectionally with metabolic and PI3K/AKT programs in liver cancer [56]. Notably, propranolol’s pharmacology, non-selective β-adrenergic antagonism, attenuates the Gs-coupled cAMP/PKA tone that feeds forward into MAPK and PI3K signaling, providing a mechanistic bridge between the enrichment readouts and the drug’s mode of action [57]. Hence, the results of this study demonstrate that although multiple signaling pathways may contribute to the anti-HCC effects of Propranolol, the PI3K–AKT signaling pathway is particularly significant. This pathway encompasses a larger number of propranolol-associated anti-HCC core targets and exerts a direct influence on proliferative and cell cycle–driven oncogenic processes central to HCC pathogenesis (Fig 9). Accordingly, the PI3K–AKT signaling pathway is the principal axis modulated by propranolol in the therapeutic management of HCC.

Extra-precision Glide docking analyses demonstrated the binding affinities of propranolol toward several kinase targets related to HCC. Propranolol exhibited markedly favorable binding energetics with JAK2, ERBB2, EGFR, and CDK2, indicative of strong molecular interactions at these active sites. In contrast, weaker binding affinities were observed for SRC, CCND1, and CDK4, as reflected by their lower binding energies and correspondingly reduced ligand efficiencies. Structural analysis of the interaction landscape revealed critical hydrogen-bonding networks involving catalytic residues, such as ARG-980 in JAK2, PHE-864 in ERBB2, and the hinge-region diad LEU-788 and THR-725 in EGFR, GLN-131 in CDK2, GLU-327, MET-229, LYS-242, and TYR-246 in PARP1, GLU-85 in CHEK1, GLU-141 in CDK4, and LYS-206 in SRC, indicative of precise orthosteric inhibition rather than indiscriminate surface interactions. While β-blockers have traditionally not been characterized as kinase inhibitors, propranolol’s aromatic oxygen-containing moiety demonstrated an exceptional structural congruence with the kinase adenine-binding pocket, elucidating the mechanistic basis for the observed high-affinity interactions.

The four top-ranked complexes with the lowest docking scores were subjected to 100 ns molecular dynamics simulations. The backbone RMSD values stabilized below 4.5 Å within the initial 10–15 ns, while the radius of gyration and solvent-accessible surface area profiles remained constant, signifying structural integrity without evidence of large-scale unfolding. Sustained hydrogen-bond occupancies persisting for 30–68% of the trajectory, together with favorable ligand–protein contact patterns, reinforced the docking-derived binding hypotheses and confirmed propranolol’s resilience under physiologically relevant conformational fluctuations within kinase active sites. Collectively, these structure-based findings corroborate the network and enrichment analyses, establishing that propranolol exhibits topological alignment and conformational adaptability toward key oncogenic drivers in HCC.

In addition to its kinase-inhibitory activity, propranolol may exhibit multifaceted antitumor properties. Preclinical studies for other cancer cells have demonstrated its capacity to suppress vascular endothelial growth factor secretion, impede endothelial cell migration, and inhibit tube formation, thereby disrupting angiogenic processes [14]. It further attenuates β-adrenergic-driven matrix metalloproteinase activity, thereby limiting tumor invasiveness. From an immunomodulatory perspective, propranolol reduces immunosuppressive cytokine production, restores natural killer cell cytotoxicity, and decreases PD-1 expression on tumor-infiltrating lymphocytes [58,59], an especially compelling feature in the context of PD-1, targeted immunotherapies. Consistent with these experimental insights, our KEGG pathway analysis highlighted Th1/Th2 cell differentiation and PD-L1 signaling, suggesting a potential synergistic interplay between propranolol and immune checkpoint blockade therapy.

Recent systematic reviews have corroborated the survival benefits of chronic non-selective β-blocker use in diverse solid malignancies, including HCC [60]. When integrated with our multi-target docking results and stable molecular dynamics trajectories, these population-level findings strongly support the extended anti-HCC efficacy of propranolol.

## Conclusion

Through the integration of network pharmacology and high-fidelity structure-based modeling, our findings revealed that propranolol may have the potential to target a core cluster of oncogenic kinases (JAK2, ERBB2, EGFR, and CDK2) and pivotal signaling cascades (PI3K–Akt, MAPK, NF-κB, and PD-L1) with sustained binding stability thus demonstrate the therapeutic benefits in HCC. This convergence of computational and predicted mechanistic data positions propranolol as a premier drug-repurposing candidate, arguably the most compelling non-oncology agent, for incorporation into combination regimens designed to overcome the multifactorial resistance inherent to current HCC therapies. Targeted *in vitro* validation, pharmacokinetic refinement, and biomarker-guided clinical trials represent critical next steps toward translating these insights into measurable patient outcomes.

## Supporting information

S1 Table Software and web tools used for network pharmacology studies. (DOCX)

S2 Table Detailed information on the top nine potential anti-HCC core targets in the PDB database and grid docking parameters in molecular docking. (DOCX)

S3 Table Comparative analysis of different physiochemical properties of Propranolol, Sorafenib and Lenvatinib. (DOCX)

S1 File Data. (XLSX)

## Abbreviations

HCC: Hepatocellular carcinoma
PPI: Protein-protein interaction
GO: Gene Ontology
KEGG: Kyoto Encyclopedia of Genes and Genomes
CC: cellular components
BP: Biological Processes
MF: Molecular Functions
PPW: Protein Preparation Wizard
XP: Extra Precision
MD: Molecular dynamics
RMSD: Root Mean Square Deviation
RMSF: Root Mean Square Fluctuation
rGyr: Radius of Gyration
SASA: Solvent-Accessible Surface Area
PSA: Polar Surface Area
