# Supplementary material for: In-silico target prediction and pathway analysis of propranolol as a potential therapeutic agent for hepatocellular carcinoma
Source: PLoS One. 2026 Feb 13;21(2):e0333978. doi: 10.1371/journal.pone.0333978 (PMC12904466; doi:10.1371/journal.pone.0333978)
Supplement: S1 Table — (DOCX) [file pone.0333978.s001.docx]

**S1 Table.** Software and web tools used for network pharmacology studies.

| **Sr. No.** | **Name** | **Online Link** | **Ref.** |
| --- | --- | --- | --- |
|  | SWISS Target Prediction | <http://www.swisstargetprediction.ch/> | ^1^ |
|  | GeneCards^®^: The Human Gene Database | <https://www.genecards.org/> | ^2^ |
|  | VENNY (2.1) | <https://bioinfogp.cnb.csic.es/tools/venny/> | ^3^ |
|  | STRING (12.0) | <https://string-db.org/> | ^4^ |
|  | Cytoscape (3.10.3) | <https://cytoscape.org/> | ^5^ |
|  | DAVID Bioinformatics | <https://davidbioinformatics.nih.gov/summary.jsp> | ^6^ |
|  | The GEPIA database | <http://gepia.cancer-pku.cn/> | ^7^ |
|  | Bioinformatics platform | <https://www.bioinformatics.com.cn/> | ^8^ |
|  | Schrödinger Suite (Maestro 14.4) | <https://www.schrodinger.com/> | ^9^ |
|  | PyMOL (3.1.1) | <https://www.pymol.org/> | ^10^ |
|  | Protein Data Bank | <https://www.rcsb.org/> | ^11^ |
|  | NCBI: PubChem | <https://pubchem.ncbi.nlm.nih.gov/> | ^12^ |
|  | Desmond (2020.1) | <https://www.deshawresearch.com/research.html> | ^13^ |

**References**

1. Daina A, Michielin O, Zoete V. SwissTargetPrediction: updated data and new features for efficient prediction of protein targets of small molecules. *Nucleic acids research.* 2019;47(W1):W357-W364.

2. Rebhan M, Chalifa-Caspi V, Prilusky J, Lancet D. GeneCards: integrating information about genes, proteins and diseases. *Trends in genetics: TIG.* 1997;13(4):163-163.

3. Oliveros JC. VENNY. An interactive tool for comparing lists with Venn Diagrams. [*http://bioinfogp*](http://bioinfogp) *cnb csic es/tools/venny/index html.* 2007.

4. Mering Cv, Huynen M, Jaeggi D, Schmidt S, Bork P, Snel B. STRING: a database of predicted functional associations between proteins. *Nucleic acids research.* 2003;31(1):258-261.

5. Lopes CT, Franz M, Kazi F, Donaldson SL, Morris Q, Bader GD. Cytoscape Web: an interactive web-based network browser. *Bioinformatics.* 2010;26(18):2347-2348.

6. Sherman BT, Hao M, Qiu J, et al. DAVID: a web server for functional enrichment analysis and functional annotation of gene lists (2021 update). *Nucleic acids research.* 2022;50(W1):W216-W221.

7. Tang Z, Li C, Kang B, Gao G, Li C, Zhang Z. GEPIA: a web server for cancer and normal gene expression profiling and interactive analyses. *Nucleic acids research.* 2017;45(W1):W98-W102.

8. Tang D, Chen M, Huang X, et al. SRplot: A free online platform for data visualization and graphing. *PloS one.* 2023;18(11):e0294236.

9. Khan SA. Network pharmacology and in silico analysis reveal Kochiae Fructus as a potential therapeutic against atopic dermatitis through immunomodulatory pathway interactions. *PloS one.* 2025;20(4):e0320818.

10. Schrodinger L. The PyMOL molecular graphics system. *Version.* 2015;1:8.

11. Berman HM, Westbrook J, Feng Z, et al. The protein data bank. *Nucleic acids research.* 2000;28(1):235-242.

12. Kim S, Chen J, Cheng T, et al. PubChem 2025 update. *Nucleic acids research.* 2025;53(D1):D1516-D1525.

13. Release S. 4: Desmond molecular dynamics system. *DE Shaw Research, New York, NY.* 2017.
