# Supplementary material for: In-silico target prediction and pathway analysis of propranolol as a potential therapeutic agent for hepatocellular carcinoma
Source: PLoS One. 2026 Feb 13;21(2):e0333978. doi: 10.1371/journal.pone.0333978 (PMC12904466; doi:10.1371/journal.pone.0333978)
Supplement: S2 Table — (DOCX) [file pone.0333978.s002.docx]

**S2 Table.** Detailed information on the top nine potential anti-HCC core targets in the PDB database and grid docking parameters in molecular docking.

| **Target** | **PDB ID** | **Method** | **Resolution** | **Grid Centre**  **(x, y, z)** | **Inner box**  **(x, y, z)** | **Outer box**  **(x, y, z)** |
| --- | --- | --- | --- | --- | --- | --- |
| SRC | 1A08 | X-RAY DIFFRACTION | 2.20 Å | 40.14, 7.52, 20.35 | 17, 10, 15 | 35.74, 28.74, 33.74 |
| EGFR | 1XKK | X-RAY DIFFRACTION | 2.40 Å | 17.78, 33.88, 37.99 | 16, 15, 15 | 35.25, 34.25, 34.25 |
| CCND1 | 6P8E | X-RAY DIFFRACTION | 2.30 Å | 39.36, 8.48, 45.31 | 10, 10, 10 | 24.4, 24.4, 24.4 |
| JAK2 | 2B7A | X-RAY DIFFRACTION | 2.00 Å | 113.62, 65.07, 9.02 | 10, 10, 10 | 22.93, 22.93, 22.93 |
| ERBB2 | 3PP0 | X-RAY DIFFRACTION | 2.25 Å | 16.67, 15.96, 27.09 | 10, 18, 10 | 26.84, 34.84, 26.84 |
| PARP1 | 2RCW | X-RAY DIFFRACTION | 2.80 Å | -23.74, 64.13, 27.22 | 12, 10, 10 | 27.07, 25.07, 25.07 |
| CDK4 | 6P8E | X-RAY DIFFRACTION | 2.30 Å | 24.07, 21.12, 73.06 | 10, 10, 10 | 34.55, 34.55, 34.55 |
| CDK2 | 1G5S | X-RAY DIFFRACTION | 2.61 Å | 13.0, -8.83, 11.31 | 13, 13, 10 | 29.04, 29.04, 26.04 |
| CHEK1 | 2AYP | X-RAY DIFFRACTION | 2.90 Å | 6.54, -2.94, 17.44 | 16, 10, 10 | 32.73, 26.73, 26.73 |
