# Supplementary material for: In-silico target prediction and pathway analysis of propranolol as a potential therapeutic agent for hepatocellular carcinoma
Source: PLoS One. 2026 Feb 13;21(2):e0333978. doi: 10.1371/journal.pone.0333978 (PMC12904466; doi:10.1371/journal.pone.0333978)
Supplement: S3 Table — (DOCX) [file pone.0333978.s003.docx]

**S3 Table.** Comparative analysis of different physiochemical properties of Propranolol, Sorafenib and Lenvatinib.

| **Property** | **Sorafenib** | **Lenvatinib** | **Propranolol** | **Implication for drug performance** |
| --- | --- | --- | --- | --- |
| **Molecular formula** | C_21_H_16_ClF_3_N_4_O_3_ | C_21_H_19_ClN_4_O_4_ | C_16_H_21_NO_2_ | Propranolol has simple structure |
| **Molecular weight** | 464.82 | 426.85 | 259.34 | Propranolol’s lower MW favours better oral bioavailability and permeability (Lipinski’s Rule of Five). |
| **Heavy atoms** | 32 | 30 | 19 | Fewer heavy atoms in Propranolol suggest improved pharmacokinetics and reduced metabolic complexity. |
| **Rotatable bonds** | 9 | 8 | 6 | Propranolol’s rigidity reduces entropy loss upon binding, improving binding affinity. |
| **H-Bond acceptors** | 7 | 5 | 3 | Fewer acceptors in Propranolol suggest a more compact and efficient hydrogen bonding profile. |
| **H-Bond donors** | 3 | 3 | 2 | Fewer Propranolol’s H-bond donors, favours membrane permeability over solubility. |
| **Molecular refractivity** | 112.48 | 112.86 | 78.44 | Lower refractivity in Propranolol suggests favourable ligand-target interactions. |
| **TPSA** | 92.35 | 115.57 | 41.49 | TPSA values are within the optimal range (≤140 Å²) for cell membrane permeability and bioavailability. |
